# Early olfactory dysfunction in experimental autoimmune encephalomyelitis reflects transient brain barrier breach and initiation of neuroinflammation in the olfactory bulb

**DOI:** 10.3389/fncel.2025.1656777

**Published:** 2025-09-03

**Authors:** Andjela Stekic, Milorad Dragic, Ivana Stevanovic, Marina Zaric Kontic, Marija Adzic Bukvic, Sanja Dacic, Milica Ninkovic, Nadezda Nedeljkovic

**Affiliations:** 1 Center for Translational Neuroscience, Department of General Physiology and Biophysics, Faculty of Biology, University of Belgrade, Belgrade, Serbia; 2 Department of Molecular Biology and Endocrinology, Vinča Institute of Nuclear Sciences-National Institute of the Republic of Serbia, University of Belgrade, Belgrade, Serbia; 3 Medical Faculty of Military Medical Academy, University of Defense, Belgrade, Serbia

**Keywords:** MS/EAE, olfactory bulb, olfactory dysfunction, subarachnoid space, CNS barriers, adenosine signaling

## Abstract

Olfactory dysfunction is increasingly recognized as an early, non-motor manifestation of multiple sclerosis (MS), but the mechanisms underlying its occurrence remain unclear. Using the rat model of experimental autoimmune encephalomyelitis (EAE), we investigated the temporal relationship between olfactory impairment, neuroinflammation, barrier integrity, and adenosine signaling in the olfactory bulb (OB) in the early stage of EAE. The study showed that more than two-thirds of EAE animals exhibited significant deficits in the buried food test as early as 3 days post-immunization (dpi), which preceded the first motor symptoms by several days. Open field test confirmed that these olfactory deficits were not due to impaired locomotion. Transient breach to the OB tissue barrier was demonstrated at 3–5 dpi by increased FITC-dextran penetration and peripheral monocyte/macrophage infiltration into the lateral aspect of the OB. The breach coincided with activation of microglia in the outer nerve layer on the lateral aspect of the OB. Oxidative stress, including elevated malondialdehyde, nitric oxide, and superoxide ion levels along with a depleted antioxidant defense system, indicated a redox imbalance, while a transient increase in neurofilament light chain serum levels at 3 dpi indicated acute neuroaxonal injury and barrier disruption at early stage EAE. At the molecular level, the simultaneous upregulation of CD73 and adenosine A_1_/A_2A_ receptors along the pial surface and in the olfactory nerve layer suggested enhanced adenosine signaling in early barrier modulation. Spatial mapping of FITC-dextran penetration, peripheral infiltrates, and microglia activation indicated access of immune cells from the subarachnoid space into the OB parenchyma. Overall, these results demonstrate that the OB is a permissive entry zone for autoreactive immune cells in the OB in early stages of EAE, highlighting olfactory and behavioral testing as promising tools for early detection and monitoring of MS.

## 1 Introduction

Multiple sclerosis (MS) is a progressive autoimmune disease in which autoreactive CD4^+^ T cells and peripheral monocytes/macrophages infiltrate the central nervous system (CNS) and cause neuroinflammation, demyelination, and axonal damage. These histopathologic changes manifest clinically over time as progressive deficits in motor function and cognitive impairment (McGinley et al., 2021; Kappos et al., 2013). Experimental autoimmune encephalomyelitis (EAE) faithfully reproduces key features of MS, including CD4^+^ T-cell-mediated neuroinflammation, myelin loss, and hindlimb paralysis and is widely recognized as a preclinical model (Procaccini et al., 2015). While the blood–brain barrier (BBB) is considered the main route for immune cell entry into the CNS (Zheng et al., 2022), there is increasing evidence that the choroid plexus (CP) and subarachnoid space (SAS), which form the cerebrospinal fluid-blood barrier, are initial portals of entry for immune cells into the CNS in EAE (Brown and Sawchenko, 2007; Saunders et al., 2008).

While the overt clinical picture in MS/EAE is dominated by motor deficits, sensory impairments often occur in the prodromal phase, long before irreversible damage occurs. Olfactory dysfunction in particular, along with visual, tactile, and auditory deficits, is one of the most common manifestations observed retrospectively in MS patients (Kim et al., 2018; Lucassen et al., 2016; Mirmosayyeb et al., 2022) and correlates strongly with the severity of the disease (Todd et al., 2023). As early deterioration of olfaction is also a hallmark of Parkinson’s, Alzheimer’s, and Lewy body diseases (Slabik and Garaschuk, 2023), elucidating the underlying mechanisms could lead to a deeper understanding of various neurodegenerative diseases, including MS. In some cases, olfactory dysfunction is unique in disease onset while other sensory systems are spared (Rey et al., 2018), raising the possibility that the olfactory system is particularly vulnerable to some neurological disorders.

Olfactory dysfunction in MS follows a temporal pattern characterized by an increased odor detection threshold in the early inflammatory phase of the disease and impaired odor discrimination emerging in full-blown disease (Lutterotti et al., 2011). This progression likely reflects a spatial gradient of neuroinflammation along the olfactory pathway, from the peripheral olfactory nerve and olfactory bulb (OB), responsible for initial detection and processing, to the higher-order cortical centers responsible for fine discrimination. Consistent with clinical observations, EAE animals also develop marked olfactory dysfunction that parallels disease progression (Kim et al., 2018; Stekic et al., 2024). The lack of robust adult neurogenesis and disturbances in the rostral migratory stream (Tepavčević et al., 2011) further exacerbates olfactory decline in neurodegenerative conditions. In our recent study, we found that the peak of EAE coincides with massive CD4^+^ T-cell and monocyte/macrophage infiltration, marked microgliosis, and elevated TNF-α, IL-1β, and IL-6 levels in the olfactory bulb (Stekic et al., 2024). Taken together, these findings emphasize a stepwise neuroinflammatory assault beginning in the sensory periphery and spreading centrally that underlies both early olfactory impairment and later discrimination failures in MS and its animal models.

Considering that olfactory dysfunction occurs at the earliest stages of MS/EAE, this study aims to determine whether the early stage of EAE is associated with the breakdown of tissue barriers and the infiltration of peripheral immune cells into the OB and how these processes lead to olfactory dysfunction. By identifying the specific inflammatory changes that occur in the OB at this critical time point, we seek to uncover the pathophysiologic mechanisms underlying MS. Understanding these early events enables a deeper understanding of MS-related sensory impairments and also paves the way for the development of biomarkers for preclinical detection of the disease.

Since olfactory deficits occur early in MS/EAE, this study aims to investigate whether barrier disruption and peripheral immune-cell entry into the OB underlie this early sensory loss. By mapping FITC-dextran leakage, immune infiltrates, and oxidative changes at 3 dpi, before motor signs emerge, the study aims to identify the triggering inflammatory events in the OB that cause olfactory dysfunction.

## 2 Materials and methods

### 2.1 Animals

For this study, 2-month-old male Dark Agouti (DA) rats (RRID: RGD_21409748) were used, purchased from the animal facility of the Military Medical Academy, University of Defense, Serbia. All experimental procedures adhered to the ARRIVE guidelines and were conducted in compliance with EU Directive 2010/63/EU. Ethics approval was granted by the Ethics Committee for the Care and Use of Laboratory Animals, Faculty of Biology, University of Belgrade (approval number: 119–01-4/11/2020–09). Animals were housed under standard conditions, including a 12-h light/dark cycle, a constant ambient temperature of 22 ± 2°C, and controlled humidity. All experimental conditions were maintained consistently across study groups to minimize performance bias.

A total of 90 animals were used in the study. Animals were randomly assigned in two experimental groups: 68 animals were assigned to the EAE group, while 22 animals formed the control group (Supplementary Table S1).

### 2.2 Induction of EAE and neurological assessment

Experimental autoimmune encephalomyelitis was induced by intradermal injection of an encephalitogenic emulsion, prepared by mixing spinal cord homogenate (1 μL phosphate-buffered saline (PBS)/1 mg tissue) with Complete Freund’s adjuvant (1 mg/mL) enriched with 4 mg/mL *Mycobacterium tuberculosis* in a 1:1 volume ratio, as previously described (Stekic et al., 2024; Dragić et al., 2021). Prior to immunization, rats were anesthetized via intraperitoneal injection of ketamine (50 mg/kg) and xylazine (10 mg/kg). Age-matched control rats remained completely intact without any injections.

Body weight and neurological scores were monitored daily using the clinical EAE severity scale for motor disability: 1—limp tail, 1.5—clumsy gait, 2—mild monoparesis or paraparesis, 3—hind paw paralysis, 3.5—paraplegia and/or quadriparesis, 4—quadriplegia, 5—moribund state or death. In parallel, animals were tested for olfactory abilities using a buried food test (BFT).

Animals were sacrificed 3, 7, or ~12 days after immunization (dpi). These time points were chosen based on olfactory testing and EAE motor scoring, as dpi when animals present olfactory dysfunction (~ 3 dpi), slight (~7 dpi), and severe (~12 dpi) motor impairment. Specifically, 3 dpi represents the pre-onset phase of EAE, occurring prior to the appearance of any motor symptoms, whereas 12 dpi corresponds to the late symptomatic stage, characterized by prominent clinical manifestations. These time points were selected to investigate the temporal relationship between olfactory dysfunction and the progression of EAE-related pathology. Criterion for olfactory impairment was a > 2-fold increase in latency in BFT compared to the baseline determined before immunization. Criteria for slight and severe motor impairments were EAE clinical score of 0.5 and 3, which corresponded to floppy tail and hind-paw paralysis, respectively. For the purpose of BBB permeability testing, additional group of EAE animals was sacrificed at 5 dpi. Animals were decapitated using the guillotine for small animals (Harvard Apparatus Co., Massachusetts, United States).

### 2.3 Buried food test

Olfactory performance was analyzed by a buried food test as previously described (Stekic et al., 2024). The test was performed daily, starting at −2 dpi until sacrifice. In brief, after the habituation phase, animals (*n* = 12) were placed in clean test arena (box 50 cm × 50 cm × 50 cm) lined with 3-cm thick bedding, with a food pellet buried 0.5 cm below the bedding in random corner of the test arena. The activity was recorded for up to 5 min under dim light (>100 lux) using ANY-MAZE camera, and the latency in finding buried food was recorded each day.

The recorded material was analyzed after each measurement and for each animal—the average latency to find buried food, determined from −2 dpi to 0 dpi, was referred to as the baseline latency (s). The time taken by an animal to find buried food from 1 dpi afterward was expressed relative to its baseline latency. The threshold for olfactory impairment was >2-fold increase compared to baseline latency. Animals that did not show typical exploratory behavior before immunization in BFT were excluded from the study. Blinding of the experimental procedure was not necessary as the test was performed on the same animals before and after the induction of EAE, and the results were analyzed using the ANY-MAZE software. The BFT was performed several times on different groups of animals.

### 2.4 Open field test

Open field test (OFT) was used to demonstrate EAE-induced changes in spontaneous locomotor activity and anxiety-related emotional behavior, as previously described (Stekic et al., 2024). Animals in the EAE group (*n* = 12) were tested at −1 dpi and at 3 dpi. Animal was placed in the center of testing black arena (100 × 100 × 50 cm), and the activity was recorded under dim light (~100 lux) for 10 min using ANY-MAZE camera. All parameters were analyzed using ANY-maze Video Tracking System 7.11. As with the BFT, blinding of the experimental procedure was not necessary as the test was performed on the same animals before and after immunization, and the results were processed using the ANY-MAZE software.

### 2.5 Immunohistochemistry and quantification

EAE animals at 3 dpi and control group (*n* = 4/group) were decapitated, and brains were dissected on ice, fixed in 4% paraformaldehyde/0.1 M phosphate buffer (pH 7.4) for 24 h, and dehydrated/cryoprotected in 10, 20, and 30% sucrose solution/0.2 M phosphate buffer. Serial 25-μm coronal brain sections (7.56–6.60 mm from bregma) were cut on cryostat, mounted on SuperFrost™ Plus adhesion microscope slides (Epredia Inc., United States), air-dried at room temperature for 1–2 h, and stored at −20° C until use.

Sections were tempered for 30 min at room temperature (RT) and immersed in PBS (pH 7.4) 2 × 5 min for rehydration. Sections were incubated with 0.3% hydrogen peroxide/methanol for 20 min and rinsed in PBS 2 × 5 min and incubated with 0.1% Triton-X/PBS for 10 min to permeabilize cell membranes. Sections were then washed 2 × 5 min in PBS and incubated with 5% normal donkey serum/PBS for 1 h to block non-specific binding of primary antibodies (Table 1). After the blocking stage, sections were incubated with primary antibodies overnight at 4° C. The next day, sections were rinsed in PBS 3 × 5 min and incubated with secondary antibodies for 2 h at room temperature. After washing in PBS for 3 × 5 min, sections were stained with 3,3-S-diaminobenzidine tetrachloride (DAB Kit, Abcam, United Kingdom), which was used as a chromogen for HRP-conjugated secondary antibodies. After stopping the reaction in PBS, sections were counterstained by incubating with hematoxylin–eosin solution for 15 s, dehydrated in 50, 75, 95, and 100% ethanol for 5 min each, cleared in xylene 2 × 5 min, and mounted with DPX medium (Sigma Aldrich, United States). Digital images (2088 × 1,550 pixels) were acquired using LEITZ DM RB light microscope (Leica Mikroskopie & Systems GmbH, Wetzlar, Germany), LEICA DFC320 CCD camera (Leica Microsystems Ltd., Heerbrugg, Switzerland), and LEICA DFC Twain software (Leica, Germany).

**Table 1:** List of primary and secondary antibodies used.

Epitope	Source and clonality	Dilution and application	Manufacturer	RRID
GFAP	Chicken, *pc*	1:500^IHC^	Novus Biologicals	RRID: AB_1556315
Iba1	Goat, *pc*	1:400^IHC^	Abcam, ab5076,	RRID: AB_2224402
CD73	Rabbit, *pc*	1:1500^WB^ 1:300^IHC^	Cell Signalling Technology Ectonucleotidases-ab-com	RRID: AB_2716625 rNu-9L (I4, I5)
A_1_R	Rabbit, *pc*	1:1000^WB^ 1:200^IHC^	Alomone	RRID: AB_2039705
A_2A_R	Rabbit, *pc*	1:500^WB^ 1: 200^IHC^	Thermo Fisher Scientific	RRID: AB_2257858
GAPDH	Rabbit, *pc*	1:2000^WB^	Invitrogen	RRID: AB_2107311
Rabbit IgG (H + L)	Goat, *pc* HRP conjugated	1:30000^WB^, 1:2000^IHC^	Abcam	RRID: AB_955447
Goat IgG (pAB)	Rabbit, *pc* HRP conjugated	1:2000^IHC^	R&D	RRID: AB_562588
Chicken IgY (H + L)	Rabbit, *pc* HRP conjugated	1:2000^IHC^	Abcam, ab6753	RRID: AB_955464

Number of cells immunoreactive for Iba1^+^ and GFAP^+^ were counted in high-power micrographic images (40 ×) using the ImageJ Cell Counter plugin. The number of immunopositive cells was counted in corresponding OB layers in *n* = 7 sections per experimental group and expressed as cell density (number of cells/mm^2^).

### 2.6 Assessment of tissue barrier permeability

The integrity of the barriers in the CNS was examined by using 10 kDa fluorescein isothiocyanate-dextran (FITC-dextran, Sigma FD10S) as a low-molecular weight tracer. Animals were anesthetized with ketamine (100 mg/kg) and xylazine (10 mg/kg) and fixed on a wooden plate for the surgery protocol. Abdomen was cut in a Y-shaped manner along the *linea alba* to enable the access to the iliac vein. Using a flexible, thin 23G cannula, 3 mg/mL dextran/PBS was administrated in the vein in an appropriate volume (0.2 mL/100 g body weight), according to a previously established protocol (Viñuela-Berni et al., 2020). After 10 min of FITC-dextran circulation, animals were perfused for 5 min with PBS at a rate of 10 mL/min. After decapitation, brains were dissected and processed, and serial 25-μm coronal brain sections (7.56–6.60 mm from bregma) were cut on cryostat, mounted on SuperFrost™ Plus adhesion microscope slides (Epredia Inc., United States), air-dried at room temperature for 1–2 h, and stored at −20° C until use.

Sections were left to temper for 30 min at RT before rehydration in PBS 3 × 10 min and mounting with Mowiol^®^ and observed using a fluorescent microscope (Zeiss Axiovert, Zeiss, Germany). Images (1,024 × 1,024) were captured at 5 × magnification. The quantification of the images was expressed as integrated density of the fluorescence signal, and the graphical data show mean values of integrated density ± SEM.

### 2.7 ELISA assay

ELISA test kit (E-EL-R2536, Elabscience Biotechnology, Wuhan, China) was used to measure the concentration of neurofilament light chain (NEFL) in sera of control and EAE rats. Animals were anesthetized with ketamine (100 mg/kg) and xylazine (10 mg/kg) and fixed on a wooden plate. Abdomen was cut appropriately to enable the access to the iliac vein. Needle (21G) was gently inserted into the vein, and the 2 mL syringe was pulled slowly to collect the blood without air bubbles. Blood samples were collected in vacutainers (BD Vacutainer^®^ Clot Activator Tube, REF 368815, BD-Plymouth, United Kingdom) and then centrifuged at 3000 rpm for 15 min at RT to obtain serum, immediately stored at −80° C until use. Prior to assaying, 4 × and 16 × diluted serum samples were tested along with non-diluted serum samples to identify the optimal dilution for ELISA assay running. The results of the optimization assay showed that 4 × was the appropriate dilution for measuring the concentration of NEFL.

### 2.8 Preparation of crude membrane (P2) fraction

Crude membrane (P2) fraction was isolated from olfactory bulb tissue by using a previously established protocol (Gray and Whittaker, 1962). In brief, olfactory bulb tissue was homogenated in ice-cold isolation buffer (0.32 M sucrose in 5 mM HEPES pH 7.4) supplemented with 0.5% w/v protease inhibitor cocktail (ab65621, Abcam, United Kingdom), in a volume ratio of 1 mg tissue/9 μL isolation buffer. Crude nuclear (P1) fraction was pelleted by centrifugation at 1,500 × g for 10 min, while centrifugation of the resulting supernatant at 16,000 × g for 30 min yielded P2 fraction in the pellet. P2 pellet was resuspended in appropriate volume of HEPES buffer. Protein concentration was determined using the Micro BCA Protein Assay Kit (Thermo Fisher Scientific, Rockford, United States). Samples were aliquoted and stored at −80°C until use.

### 2.9 Western blot analyses

P2 fraction samples were adjusted to 1 μg/μl in 6 × Laemmli buffer [4% sodium dodecyl sulfate (SDS), 0.02% bromophenol blue, 20% glycerol, and 125 mmoL/L Tris–HCl] and boiled at 95°C for 5 min. Ten microliter aliquots of P2 samples were loaded onto 10% SDS-polyacrylamide gels, and electrophoresis was run for ~1 h 30 min at the voltage of 100 V. Samples were electrotransferred to 0.45 μm PVDF support membranes (Millipore, Germany) using the Trans-Blot Turbo System (Bio-Rad, Hercules, CA, United States). Membranes were blocked in 5% non-fat dry milk (SERVA, Germany) in Tris-buffered saline containing 0.1% Tween-20 (TBST) for 1 h at RT. Support membranes were incubated with primary antibodies diluted in TBST (Table 1) overnight at 4°C. Membranes were rinsed 3 × 5 min in PBS and incubated with HRP-conjugated secondary antibodies diluted in TBST (Table 1) for 2 h at RT. After one more round of washing in PBS 3 × 5 min, chemiluminescent signal was visualized with ECL solution (Bio-Rad, Hercules, CA, United States) using ChemiDoc Imaging System (Bio-Rad Laboratories, Inc.).

Primary and secondary antibodies were removed using a mild stripping protocol (0.2 mmoL/L glycine, 0.1% SDS, and 1% Tween-20, pH 2.2) to blot glyceraldehyde 3-phosphate dehydrogenase (GAPDH), which was used as a loading control. Abundance of the target protein was quantified by densitometry using ImageJ software (NIH, Bethesda, MA, United States). Optical density (OD) of the target protein band was normalized to the OD of the GAPDH band in the same lane, and the mean ratio from four-five different OB samples per EAE group was expressed relative to the mean ratio of control (defined arbitrarily as 100%). Data were expressed as mean target protein abundance ± SD (%) of four-five samples/group blotted two times.

### 2.10 Assessment of oxidative stress

Animals of control and EAE groups (*n* = 4/group) were decapitated at 3 and 12 dpi. The OB tissue was immediately dissected on ice and homogenized (1:9 ratio) with 0.32 M sucrose in 5 mM Tris–HCl buffer at pH 7.4. Each sample was subsequently centrifuged at 1,000 × *g* for 10 min at 4°C to collect the supernatant containing the whole cell fraction. Protein concentration in the samples was determined using the Micro BCA Protein Assay Kit (Thermo Fisher Scientific, Rockford, United States), and they were kept at −80° C until use. Samples were adjusted to 1 μg/μl before oxidative measurements.

Lipid peroxidation, one of the major indices of oxidative stress, was determined by the spectrophotometric method based upon a reaction of malondialdehyde (MDA), a product of lipid peroxidation, with 0.375% thiobarbituric acid (TBA) in Tris–HCl (pH 7.4) at 100° C for 60 min (Girotti et al., 1991). The reaction resulted in a red supernatant whose absorbance was measured at 535 nm. The results were expressed as mean MDA concentration (mmol/mg protein) ± SD from *n* = 4 samples assayed in duplicate.

Content of superoxide anion radical (O^2−^) was quantified by the method based on the reduction of nitroblue tetrazolium – NBT (NBT; Merck, Darmstadt, Germany) to monoformazan by O_2_^−^ in the alkaline nitrogen saturated medium, as previously described (Zeljkovic Jovanovic et al., 2024). The resulting yellow-colored reduced product was measured spectrophotometrically at 550 nm (Ultrospec 2000). The results were expressed as nmol NBT/mg protein/min ± SD from *n* = 4 samples assayed in duplicate.

Nitrosative stress was assessed by measuring nitrite and nitrate (NO_2_^−^ + NO_3_^−^) concentrations in deproteinized samples using a spectrophotometric method at 492 nm. After transforming nitrates into nitrites by cadmium reduction (Navarro-Gonzálvez et al., 1998), nitrite level was assayed using the Griess colorimetric method, which involved the use of 1.5% sulfanilamide in 1 M HCl and 0.15% N-(1-naphthyl) ethylenediamine dihydrochloride in distilled water. Nitrite concentrations were determined based on a standard curve generated using known nitrite concentrations and expressed as mean NO concentration (μmol/mg protein) ± SD from *n* = 4 independent samples assayed in duplicate.

Total superoxide dismutase (tSOD) activity, which combines the activity of the cytosolic Cu/ZnSOD and mitochondrial MnSOD, was assayed by a spectrophotometric approach based on the measurement of the decrease in the rate of spontaneous epinephrine auto-oxidation at 480 nm (Sun and Zigman, 1978) as previously described (Adzic et al., 2017). The kinetic activity was monitored in a carbonate buffer, after the addition of 10 mM epinephrine (Sigma, St. Louis, MO, United States). The activity was expressed as units per milligram of total protein (U/mg protein), where one unit represents the amount of enzyme required to inhibit epinephrine auto-oxidation by 50%. The results are expressed as mean activity (U/mg protein) ± SD from n = 4 independent samples assayed in duplicate.

Glutathione (GSH) is a naturally occurring tripeptide and a powerful antioxidant, crucial for protecting cells from damage caused by free radicals. Total GSH content was determined by a spectrophotometric assay based on oxidation of GSH in the presence of sulfhydryl reagent 5,5′-dithio-bis(2-nitrobenzoic acid) (DTNB), resulting in the formation of the yellow derivative 5′-thio-2-nitrobenzoic acid (TNBA), which is measurable at 412 nm. The results are expressed as mean TNBA concentration (nmol/mg protein) ± SD from *n* = 4 separate determinations, performed in duplicate.

The concentration of total sulfhydryl (SH) groups in tissue homogenates was measured spectrophotometrically at 412 nm in a phosphate buffer (0.2 mol + 2 mmol EDTA, pH 9) using 5,5-dithiobis-2-nitrobenzoic acid (DTNB, 0.01 M) (Ellman, 1959). The results were expressed as mean SH (nmol/mg protein) ± SD from n = 4 independent samples assayed in duplicate.

### 2.11 Study design and statistics

A total of 90 animals were divided into 22 experimental units (4–5 animals/cage), of which 17 experimental units constituted the EAE group and 5 formed the control group (Supplementary Table S1). The sample size was estimated using G*Power analysis (Faul et al., 2007). Animals were sacrificed at 3, 5, 7, and ~12 dpi for different analyses. Samples from animals belonging to one experimental unit were not pooled and used only for one particular analysis.

Histological assessments and image quantification were performed by an investigator blinded to the experimental groups to ensure unbiased evaluation.

Quantitative data obtained within the study fell into the category of continuous data, with the exception of neurological scores, which were discrete. Data were analyzed using descriptive statistics and expressed as mean ± SD or SEM, as indicated in figure legends. The normality of data was tested using the Shapiro–Wilk test, and then, appropriate parametric or non-parametric tests were used. For EAE 3 dpi to control/−1 dpi group comparisons (OFT, GFAP, and Iba1 immunohistochemistry quantification, Western blot analyses), two-tailed Student’s *t*-test was used or Wilcoxon signed rank test if the normality condition was not met. For comparisons of measurements at 3 dpi, 7 dpi, and ~12 dpi to control, one-way ANOVA followed by Dunnett’s multiple comparisons test was used. Values of *p* < 0.05 were considered statistically significant.

**Figure 1:**
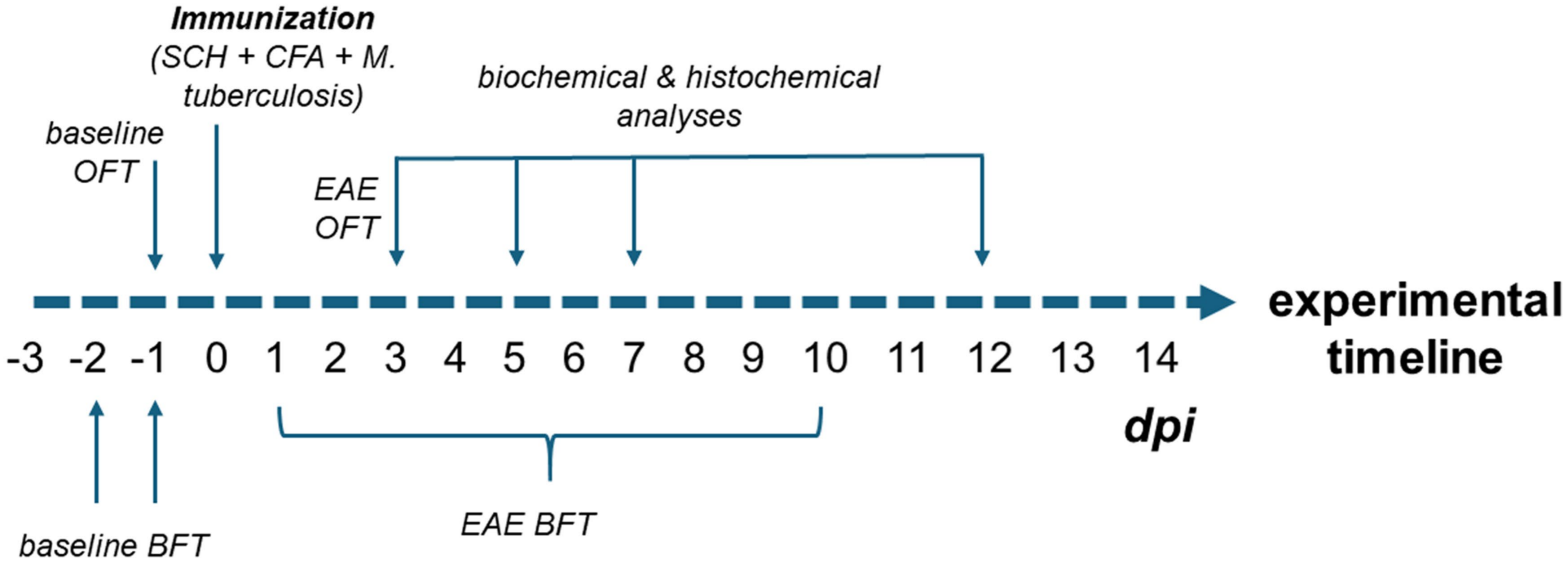
Experimental design of the study. To establish baseline olfactory function, animals were tested in the BFT on days −3, −2, and −1 prior to immunization. Baseline locomotor activity was assessed using the OFT on day −1. Following immunization (day 0), animals underwent daily BFT assessments to monitor the onset of olfactory impairment, which continued until the motor symptoms started to interfere with test performance (approximately 10 dpi). As the onset of olfactory dysfunction was observed at 3 dpi, an additional OFT was performed to rule out subtle motor deficits as a confounding factor in BFT performance. Biochemical (ELISA, oxidative stress measurements, Western blotting) and histological analyses (FITC-dextran assay, immunohistochemistry) were conducted at multiple time points: early EAE (3 and 5 dpi), motor symptom onset (7 dpi), and peak disease stage (12 dpi).

All statistical analyses and graphing were performed using the GraphPad Prism 9.0 software package (San Diego, CA). The complete experimental plan with time schedule, model, and analyses is shown in Figure 1.

## 3 Results

**Figure 2:**
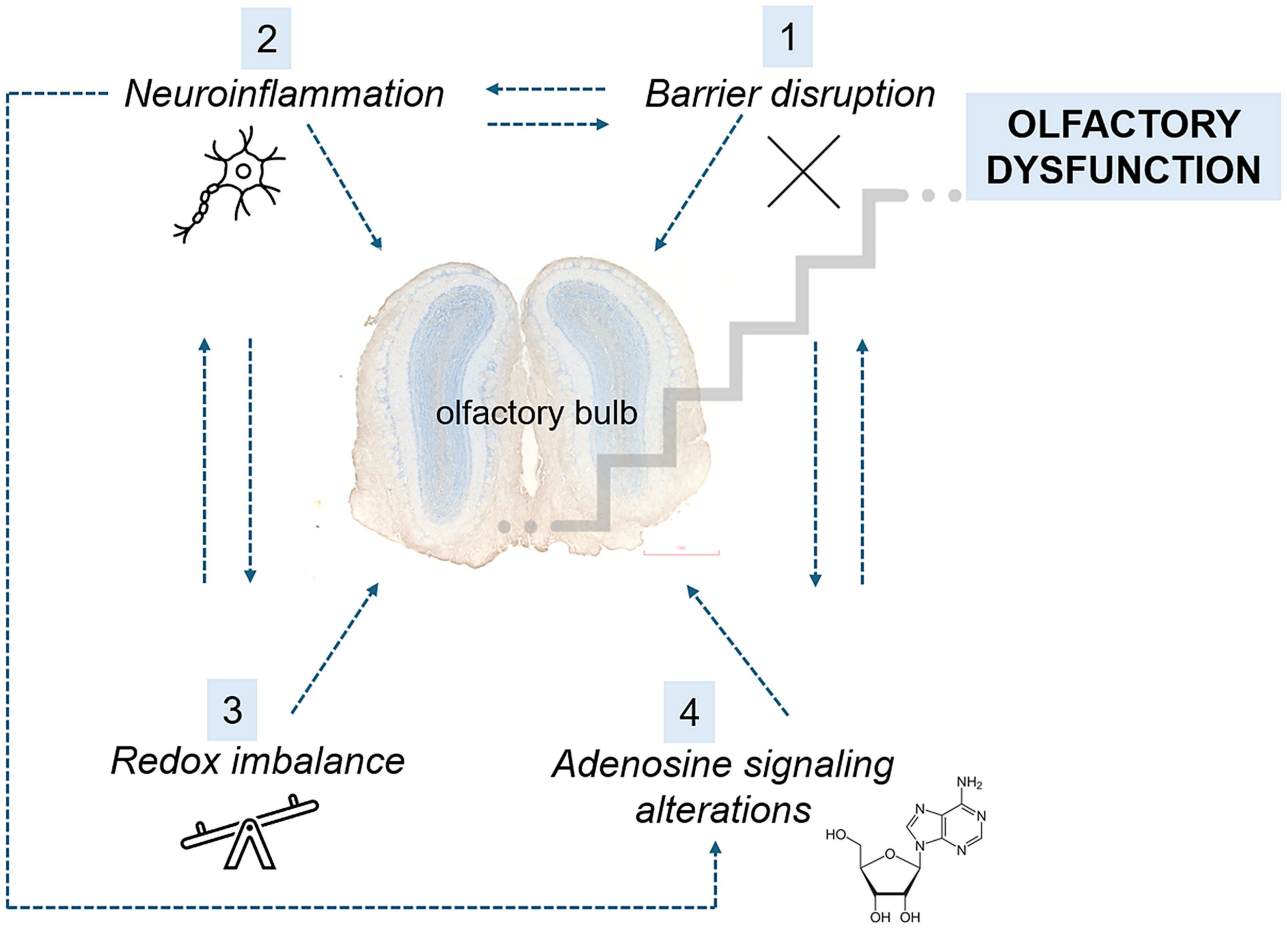
Summary of the main early changes observed after EAE induction. At the early stages of EAE, BBB disruption (1) facilitates the infiltration of peripheral monocytes into the OB, initiating a vicious cycle of neuroinflammation (2). This neuroinflammatory response further compromises BBB integrity, allowing for increased infiltration and amplifying the inflammatory cascade. Concurrently, the proinflammatory milieu leads to an imbalance between prooxidants and antioxidants (3), resulting in cellular damage and the perpetuation of neuroinflammation. Neuroinflammation also induces the upregulation of CD73, an adenosine-producing enzyme, as well as adenosine receptors A_1_ and A_2A_ (4). These changes contribute to further BBB permeability. Collectively, these events in the OB manifest behaviorally as olfactory dysfunction.

This study had two main objectives. The first was to determine whether the olfactory bulb (OB) undergoes disruption of the blood–brain or blood–CSF barriers during the early stages of EAE and whether such barrier dysfunction facilitates peripheral immune cell infiltration that contributes to olfactory impairment. The second objective was to assess whether dysregulation of adenosine signaling, given its dual role in modulating olfactory sensory processing within OB neural circuits and in maintaining CNS barrier integrity, contributes to the onset of early pathological changes and the emergence of olfactory deficits in EAE. A graphical summary of the results is shown in Figure 2.

### 3.1 Assessment of olfactory and motor impairment in EAE

**Figure 3:**
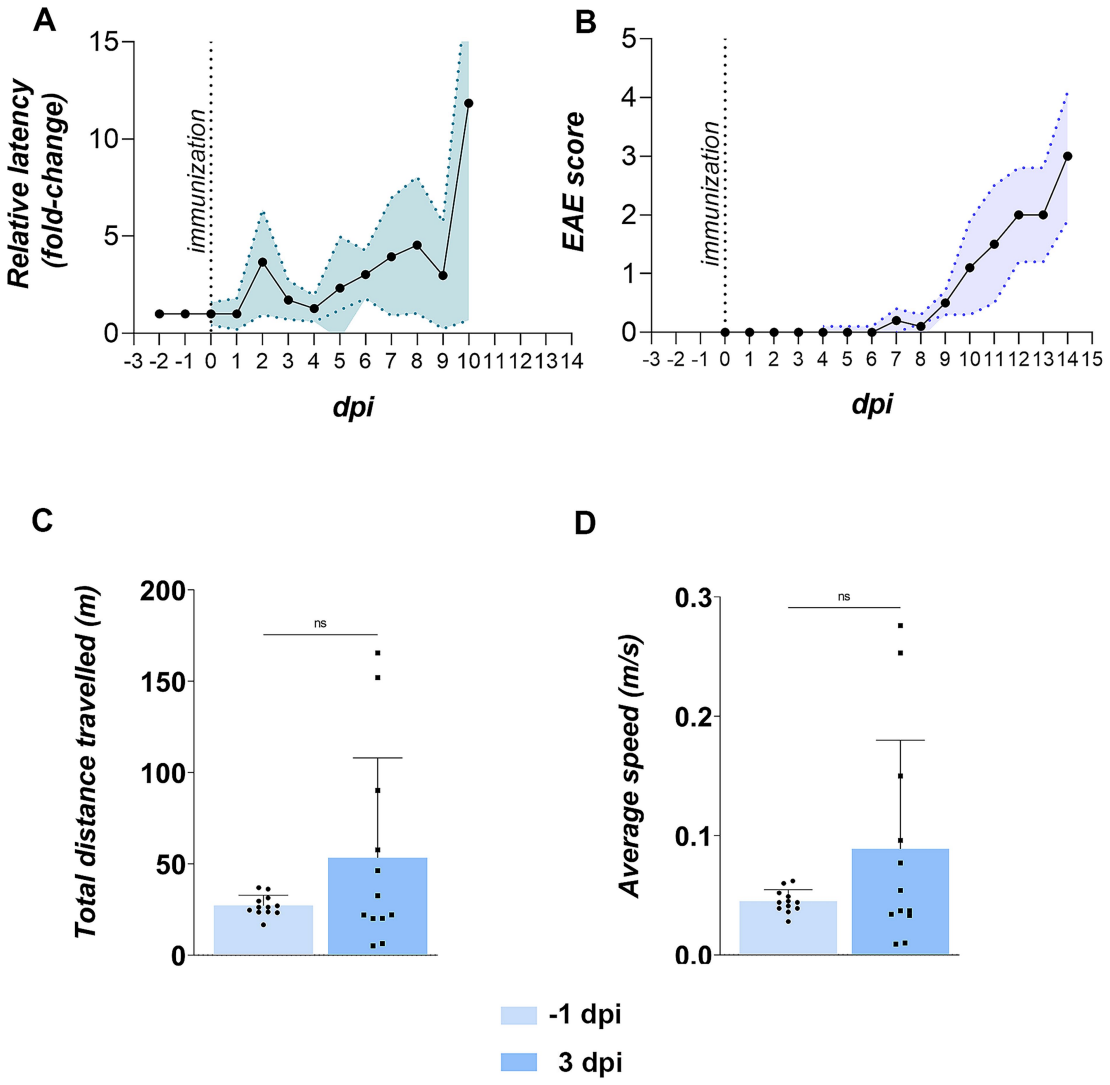
Olfactory and motor functions in EAE animals. **(A)** Buried food test (BFT) performance. The BFT was conducted daily starting from −2 days post-immunization (dpi). Latency in finding buried from −2 to 0 dpi was averaged to establish baseline latency (in seconds) for each animal. Daily performance from 1 dpi onward is shown as latency relative to baseline. Data represent the mean relative latency ± SD (shaded area) for 12 EAE animals. **(B)** EAE symptom severity. Animals were scored daily for motor signs using a standard EAE scoring scale. Data represent daily mean EAE scores ± SD (shaded area) for 12 animals. **(C,D)** Motor performance in the open field test (OFT). EAE animals were tested at −1 and 3 dpi for motor behavior. Bars represent mean values ± SD for 12 animals. Statistical significance: ns, not significant.

More than two-thirds of EAE rats (69.1%) developed olfactory dysfunction, defined as a >2-fold increase in latency to locate buried food versus baseline (Figure 3A). Deficits first appeared at ~3 dpi, abated briefly, then re-emerged by ~5 dpi, and progressively worsened through the motor phase. By contrast, the earliest motor sign, a floppy tail, was not observed until ~7 dpi, while severe motor deficits (hind-paw paralysis) only emerged by ~12 dpi (Figure 3B). These disease course kinetics establish olfactory impairment as an early, prevalent feature of EAE that precedes overt motor pathology. On the basis of these behavioral trajectories, cohorts were sacrificed at 3 dpi (early stage), with additional groups at 5 dpi, 7 dpi, and 12 dpi in some measurements to capture subsequent stages.

To rule out the possibility that impaired performance in BFT resulted from subtle motor deficits during early stage EAE, motor performance was assessed in EAE animals at −1 dpi and 3 dpi using the open field test (OFT). No significant differences were obtained in mobility parameters at 3 dpi compared to the pre-induction state, including total distance traveled (Figure 3C) and average speed (Figure 3D), indicating that prolonged BFT latency at 3 dpi was not attributable to impaired locomotion.

However, EAE rats at 3 dpi exhibited clear anxiety-like behavior. Specifically, despite unchanged average speed with an increasing trend, they showed increased freezing and immobility in the peripheral zones of the OFT arena. Moreover, they demonstrated earlier first entry into peripheral fields and corners, fewer corner entries, and elevated freezing in corner areas, collectively indicating diminished exploratory drive relative to baseline (Table 2).

**Table 2:** OFT parameters.

Parameter	−1 dpi	3 dpi
		Student’s *t-*test/Wilcoxon matched-pairs signed rank test
Number of entries in central fields	1.833 ± 1.899	1.5 ± 2.195
	(*W* = −8, *p =* 0.6758)	
Time spent in central fields (s)	10.53 ± 12.58	5.4 ± 8.298
	(*W* = −21, *p* = 0.3223)	
Distance traveled in central fields (m)	0.8349 ± 1.04	1.18 ± 1.905
	(*W* = −3, *p* = 0.9219)	
Latency to central fields entry (s)	239.1 ± 157.1	260.3 ± 173.3
	(*t* = 0.3445, *df* = 5, *p* = 0.7445)	
Average speed in central fields (m/s)	0.078 ± 0.02	0.2007 ± 0.07241
	(*W* = 19, *p* = 0.0625)	
Maximal speed in central fields (m/s)	0.32 ± 0.07	1.404 ± 1.388
	(*W* = 11, *p* = 0.3125)	
Mobile time in central fields (s)	10.5 ± 12.6	5.4 ± 8.3
	(*W* = −21, *p* = 0.3223)	
Freezing time spent in central fields (s)	0.0 ± 0.0	4.3 ± 8.2
	(*W* = 10, *p* = 0.125)	
Freezing bouts in central fields	0.0 ± 0.0	1.1 ± 2.0
	(*W* = 10, *p* = 0.125)	
Number of entries in peripheral fields	3.5 ± 2.6	11.2 ± 16.4
	(*W* = 15, *p =* 0.4785)	
Time spent in peripheral fields (s)	581.9 ± 13.8	579.0 ± 30.5
	(*W* = 18, *p* = 0.5186)	
Distance traveled in peripheral fields (m)	25.8 ± 5.7	48.0 ± 46.8
	(*W* = 28, *p* = 0.3013)	
**Latency to first entry in peripheral fields (s)**	7.1 ± 6.7	1.8 ± 1.8
	(*W* = −66, ***p* = 0.0063**)	
Average speed in peripheral fields (m/s)	0.04 ± 0.01	0.08 ± 0.09
	(*W* = 28, *p* = 0.2925)	
**Maximal speed in peripheral fields (m/s)**	0.67 ± 0.21	2.08 ± 1.34
	(*W* = 62, ***p* = 0.0122**)	
**Mobile time in peripheral fields (s)**	549.6 ± 26.9	476.9 ± 100.7
	(*W* = −58, ***p* = 0.021**)	
**Immobile time in peripheral fields (s)**	32.3 ± 20.7	102.2 ± 111.1
	(*W* = 58, ***p* = 0.021**)	
**Immobile episodes in peripheral fields**	1.9 ± 1.2	4.4 ± 3.7
	(*W* = 56, ***p* = 0.0264**)	
Freezing bouts in peripheral fields	10.4 ± 4.9	17.9 ± 15.0
	(*W* = 30, *p* = 0.1992)	
**Freezing time in peripheral fields (s)**	140.1 ± 77.6	500.5 ± 45.1
	(*t* = 20.22, *df* = 11, ***p* < 0.0001**)	
**Number of entries in corners**	27.4 ± 5.9	15.3 ± 7.4
	(*t* = 9.298, *df* = 11, ***p* < 0.0001**)	
Time spent in corners (s)	413.4 ± 36.8	401.1 ± 144.2
	(*t* = 0.2707, *df* = 11, *p* = 0.7917)	
Distance traveled in corners (m)	11.4 ± 2.8	10.7 ± 6.5
	(*t* = 0.4156, *df* = 11, *p* = 0.6857)	
**Latency to first entry in corners (s)**	16.0 ± 11.4	6.3 ± 5.3
	(*W* = −60, ***p* = 0.0161**)	
Average speed in corners (m/s)	0.027 ± 0.007	0.039 ± 0.047
	(*W* = −2, *p* = 0.9697)	
**Maximal speed in corners (m/s)**	0.579 ± 0.169	1.6 ± 1.0
	(*W* = 66, ***p* = 0.0068**)	
Mobile time in corners (s)	382.9 ± 44.3	304.5 ± 112.6
	(*t* = 1.970, *df* = 11, *p* = 0.0746)	
Immobile time in corners (s)	30.4 ± 19.9	96.6 ± 113.6
	(*W* = 50, *p* = 0.0522)	
Immobile episodes in corners	1.8 ± 1.06	4.2 ± 3.8
	(*W* = 53, ***p* = 0.0337**)	
**Freezing bouts in corners**	10.1 ± 4.9	16.5 ± 7.0
	(*t* = 2.495, *df* = 11, ***p* = 0.0298**)	
**Freezing time in corners (s)**	127.2 ± 70.1	353.5 ± 133.5
	(*t* = 5.740, *df* = 11, ***p* = 0.0001**)	
Total distance traveled (m)	27.1 ± 5.7	53.3 ± 54.6
	(*W* = 28, *p* = 0.3013)	
Average speed (m/s)	0.045 ± 0.01	0.089 ± 0.09
	(*W* = 28, *p* = 0.2925)	

Bolded terms and p-values indicate OFT parameters altered in a statistically significant manner.

### 3.2 Compromise of the blood–brain barrier integrity and immune privilege of the CNS

**Figure 4:**
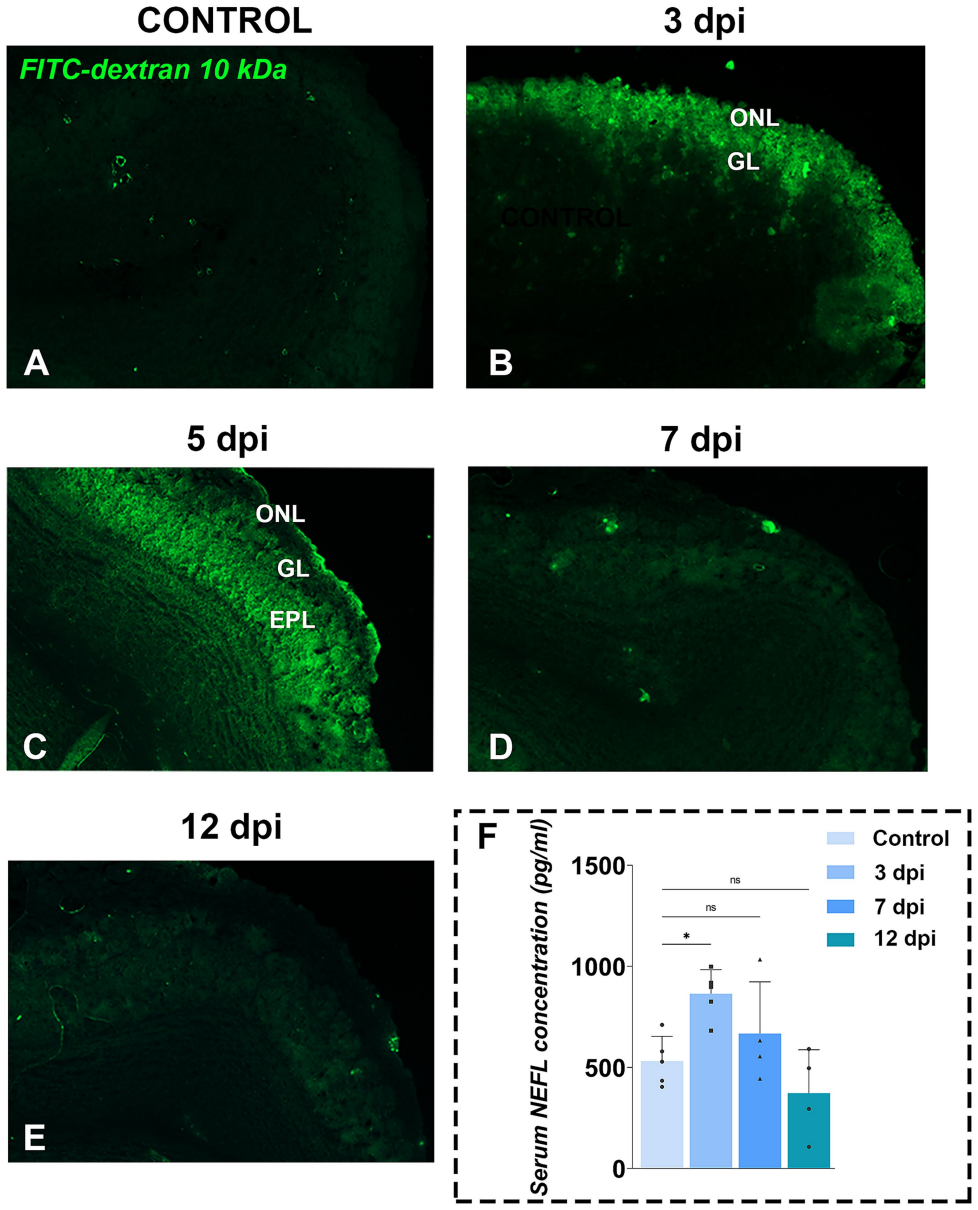
Functional tissue barrier testing in OB. **(A–E)** Representative fluorescence photomicrographs of FITC-dextran assay in OB sections from control **(A)** and EAE animals at 3 dpi **(B)**, 5 dpi **(C)**, 7 dpi **(D)**, and 12 dpi **(E)**. Green fluorescence indicates FITC-dextran penetration, suggesting compromised brain tissue barriers in the OB. Abbreviations: ONL—olfactory nerve layer; GL—glomerular layer; EPL—external plexiform layer. **(F)** Serum neurofilament light chain (NEFL) concentrations. NEFL levels were measured by ELISA in serum samples collected from EAE animals at 3, 7, and 12 dpi. Bars represent mean concentrations (pg/ml) ± SD, from *n* = 4–5 animals per time point, assayed in duplicate. Statistical significance: *p < 0.05; ns, not significant.

To assess barrier integrity at the level of the OB, we injected 10 kDa FITC-dextran and quantified its penetration into OB parenchyma (Figures 4A–E). In controls, minimal level of FITC fluorescence confirmed an intact tissue barriers (Figure 4A). By 3 dpi, the fluorescence was restricted to the ONL (Figure 4B), extending into the GL and EPL by 5 dpi (Figure 4C). Fluorescent signal returned to baseline by 7 dpi (Figure 4D) and 12 dpi (Figure 4E), indicating recovered integrity of OB barriers. Quantification of FITC-dextran fluorescence (Supplementary Figure S1), expressed as the integrated density of the fluorescence signal, confirmed the increase observed at 3 dpi [213.5 ± 10.84, *F*_(4, 105)_ = 12.99, *p* < 0.0001] and 5 dpi (232.2 ± 10.96, *p* < 0.0001), while the values at 7 dpi (161.1 ± 13.25, *p* = 0.1760) and 12 dpi (140.6 ± 23.00, *p* = 0.9721) were similar to the control values (125.5 ± 7.083). Functionally, disruption of brain barriers allows blood-borne substances and immune cells to enter the CNS while impairing clearance of neural proteins into the circulation. Consistent with FITC-dextran leakage, serum levels of neurofilament light chain (NEFL), a well-established biomarker of neuronal damage, rose transiently at 3 dpi [864.5 ± 119.0 pg./mL; *F*_(3,14)_ = 6.083, *p* = 0.0072] and then declined to control level by 7 dpi (666.5 ± 257.0 pg./mL) and 12 dpi (372.3 ± 215.6 pg./mL) (Figure 4F).

**Figure 5:**
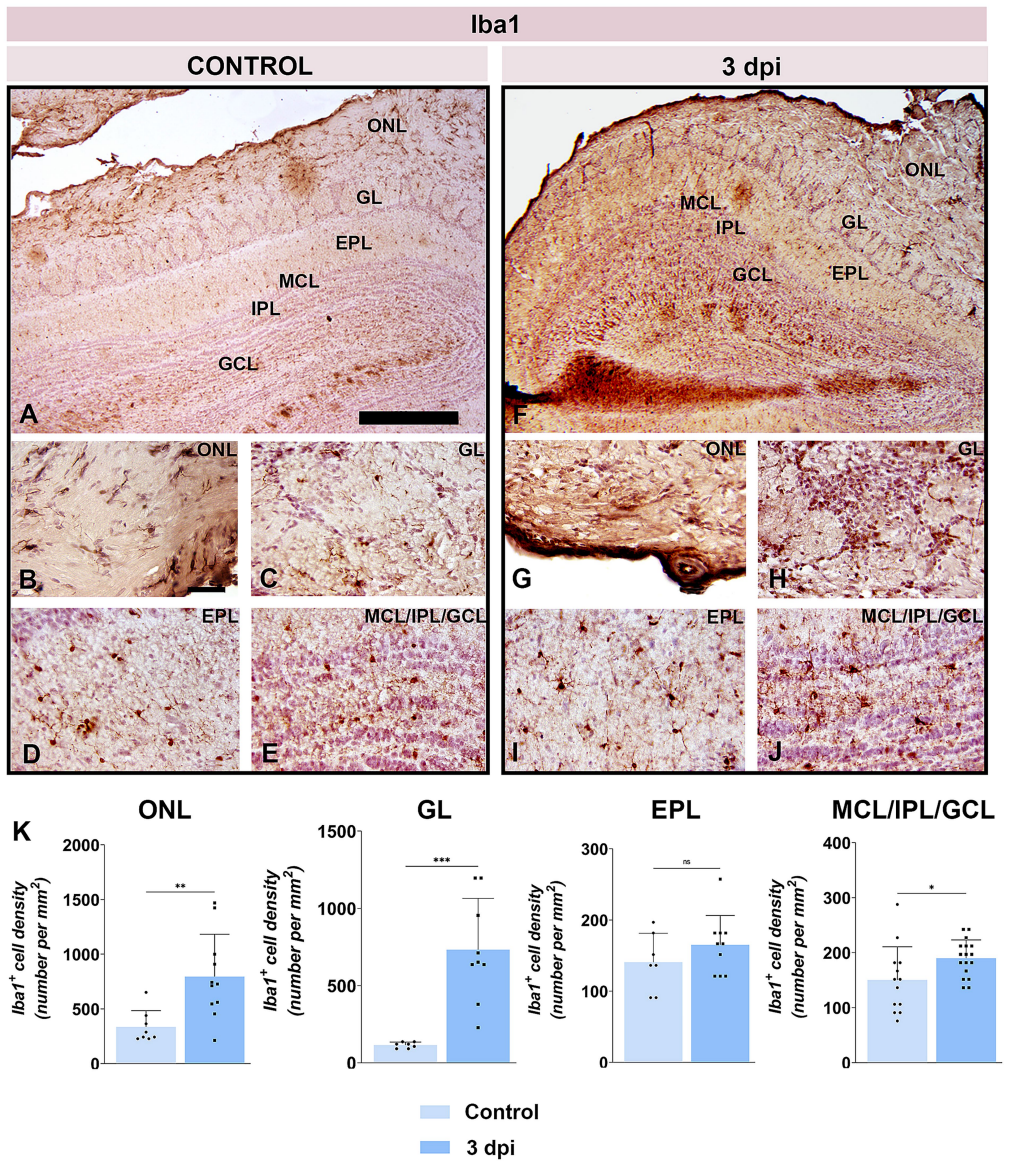
Iba1 immunolabeling of OB sections. **(A–E)** Representative Iba1-stained OB sections from control animals. **(F–J)** Representative OB sections from EAE animals at 3 dpi, showing increased Iba1^+^ cell presence. ONL, olfactory nerve layer; GL, glomerular layer; EPL, external plexiform layer; MCL, mitral cell layer; IPL, internal plexiform layer; GCL, granule cell layer. Scale bars: 500 μm **(A,F)**; 50 μm **(B–E,G–J)**. **(K)** Quantification of Iba1^+^ cell density across OB layers. Bars represent mean number of Iba1^+^ cells per mm^2^ ± SEM, based on 4 animals per group and 7 sections per animal. Statistical significance: *p < 0.05, **p < 0.01, ***p < 0.001; ns, not significant.

The breach of BBB at the OB level was further demonstrated by immunohistochemical labeling directed to ionized calcium-binding adaptor molecule 1 (Iba1), a widely used marker for microglia/macrophages immunolabeling (Figures 5A–J). At 3 dpi, numerous round Iba1^+^ cells, presumed infiltrating monocytes/macrophages, were observed along the lateral aspect of the OB and solely in the superficial layers (Figures 5G,H), a feature completely absent in control sections (Figures 5A–C). Quantification revealed a 2- to 3-fold increase in Iba1^+^ cell density in the ONL and GL (Figure 5K). In deeper OB layers, only resident microglia with mildly reactive morphology were present (Figures 5I,J), and cell density rose modestly in the MCL/IPL/GCL (*t* = 2.282, *df* = 28, *p* = 0.0303), with no significant changes in the EPL (Figure 5K).

These data confirmed compromised integrity of tissue barrier in the OB at 3 dpi, enabling peripheral immune cell infiltration and transient elevation of CNS-derived proteins in the bloodstream.

### 3.3 Assessing neuroinflammation in the OB

Pathophysiological consequences of early BBB disruption include activation of resident glia, heightened oxidative stress, and release of proinflammatory cytokines, all of which contribute to myelin injury and axonal loss. Accordingly, we evaluated key hallmarks of neuroinflammation in the OB at 3 dpi.

**Figure 6:**
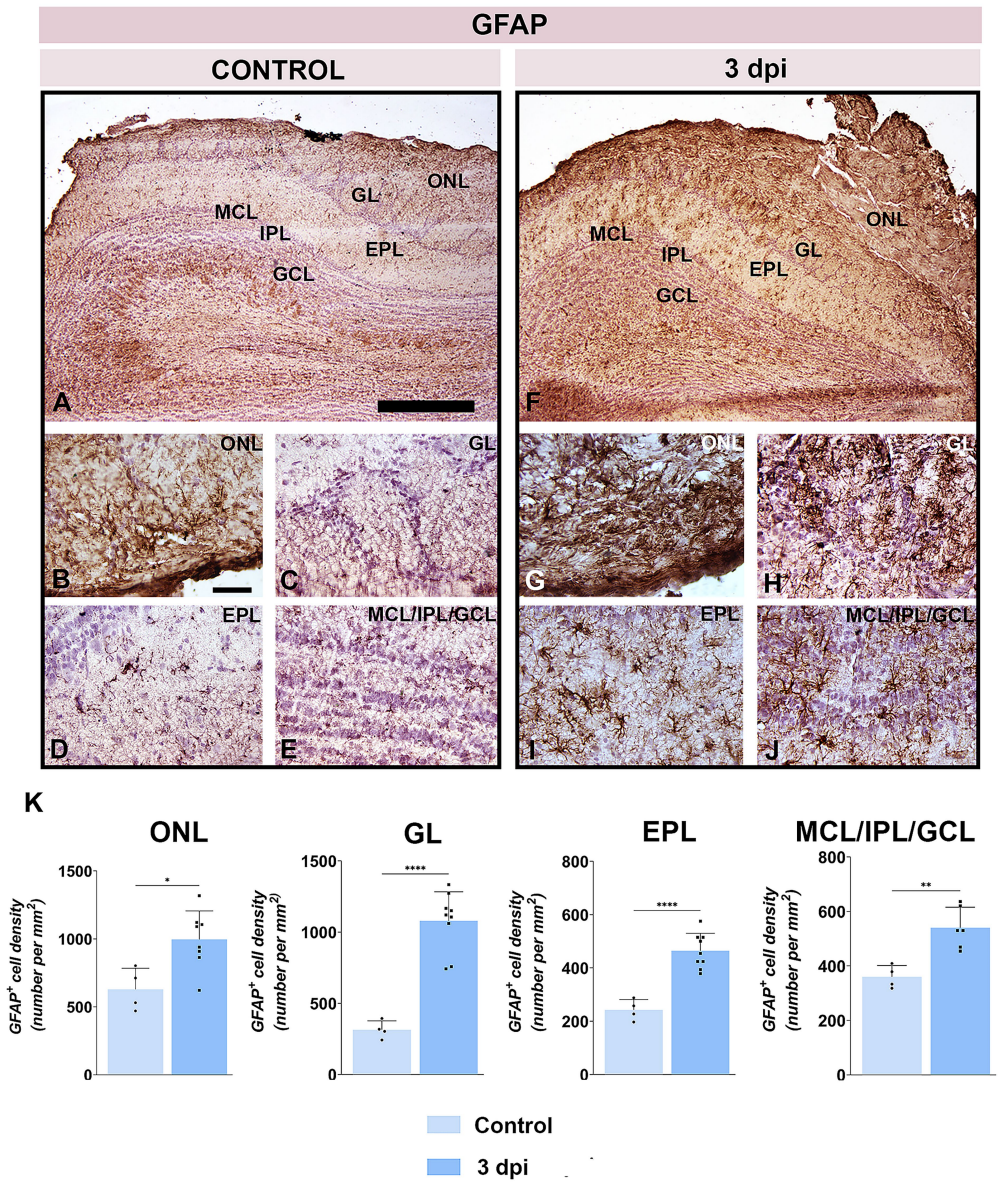
GFAP immunolabeling of OB sections. **(A–E)** Representative GFAP-stained OB sections from control animals. **(F–J)** Representative OB sections from EAE animals at 3 dpi, showing increased GFAP^+^ cell presence. ONL, olfactory nerve layer; GL, glomerular layer; EPL, external plexiform layer; MCL, mitral cell layer; IPL, internal plexiform layer; GCL, granule cell layer. Scale bars: 500 μm **(A,F)**; 50 μm **(B–E,G–J)**. **(K)** Quantification of GFAP^+^ cell density across OB layers. Bars represent mean number of GFAP^+^ cells per mm^2^ ± SEM, based on 4 animals per group and 7 sections per animal. Statistical significance: *p < 0.05, **p < 0.01, ****p < 0.0001.

Immunohistochemistry for glial fibrillary acidic protein (GFAP) showed markedly increased reactivity throughout the OB (Figures 6A–J), with staining intensity diminishing from superficial to deep layers. In the ONL, olfactory ensheathing cells (OECs) exhibited more intense GFAP immunoreactivity at 3 dpi (Figure 6G) compared to control (Figure 5B). In the GL, astrocytes were strongly GFAP-positive and hypertrophied (Figure 6H). Reactivity remained elevated in the EPL (Figure 6I) and IPL (Figure 6J) relative to controls (Figures 6D,E), although astrocytes preserved their stellate morphology and territorial domains. Quantitative analysis confirmed significant increases in GFAP^+^ cell density in the ONL, GL, EPL, and merged MCL/IPL/GCL regions at 3 dpi (Figure 6K).

**Figure 7:**
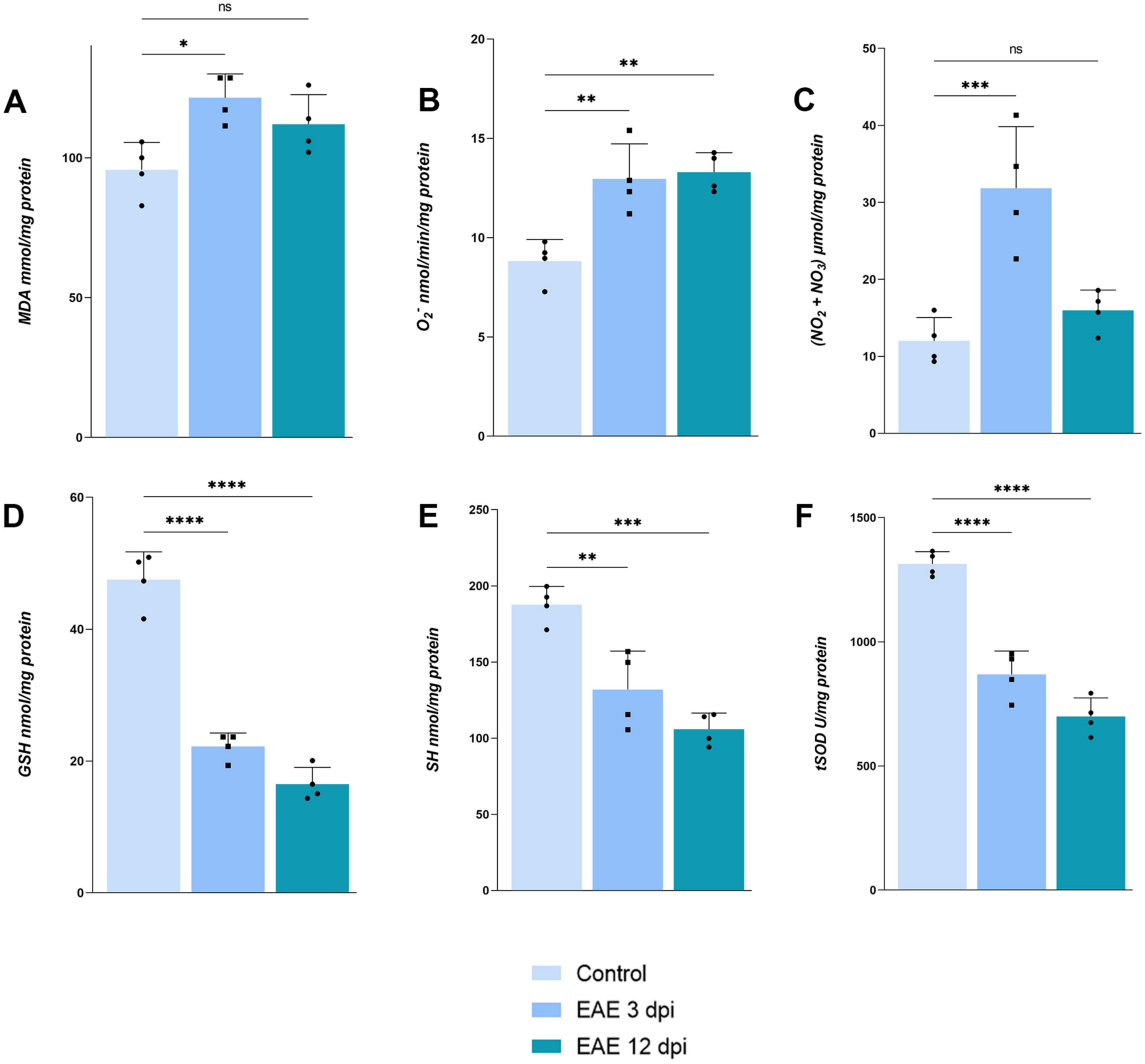
Assessment of oxidative stress parameters in OB. **(A)** Malondialdehyde, **(B)** O_2_^−^, **(C)** nitrates and nitrites, **(D)** reduced glutathione, **(E)** protein thiols, **(F)** total superoxide dismutase. Bars represent mean values in corresponding units ± SD of *n* = 4 samples per group.

Biochemical analysis of OB tissue at 3 dpi and 12 dpi revealed a pronounced, but transient oxidative imbalance (Figure 7). At 3 dpi, markers of oxidative damage: MDA [Figure 7A; 121.4 ± 8.571 nmol/mg protein, *F*_(2, 9)_ = 7.235, *p* = 0.0113], O₂^−^ [Figure 7B; 12.95 ± 1.776, *F*_(2, 9)_ = 14.06, *p* = 0.0032], and NO [Figure 7C; 31.84 ± 8.004 μmol/mg protein, *F*_(2, 9)_ = 16.47, *p* = 0.0008], were significantly elevated when compared to control MDA (95.72 ± 9.756), O₂^−^ (8.820 ± 1.084), and NO (12.00 ± 3.032), whereas antioxidant defense system, including reduced glutathione [Figure 7D; 22.22 ± 2.032 nmol/mg protein, *F*_(2, 9)_ = 114.3, *p* < 0.0001], protein thiols [Figure 7E; 132.0 ± 25.21 nmol/mg protein, *F*_(2, 9)_ = 23.35, *p* = 0.0035], and tSOD [Figure 7F; 869.0 ± 94.05 U/mg protein, *F*_(2, 9)_ = 71.63, *p* < 0.0001] activity, was markedly reduced compared to control GSH (47.49 ± 4.236), SH (187.6 ± 12.13), and tSOD (1,314 ± 49.25). Although O_2_^−^ remained elevated by 12 dpi (13.30 ± 0.9833, *p* = 0.0019), MDA (112.0 ± 10.58, *p* = 0.0945) and NO levels (15.95 ± 2.651, *p* = 0.4802) returned to baseline, mirroring the transient tissue barrier breach, whereas antioxidative defense remained impaired (GSH 16.49 ± 2.550, *p* < 0.0001; SH 105.9 ± 10.57, *p* = 0.0002; tSOD 699.2 ± 74.87, *p* < 0.0001). These data indicate that early overproduction of pro-oxidative markers drives oxidative stress in the OB but surprisingly does not persist into advanced disease stages.

Together, these data demonstrate that barrier breach and peripheral immune cell entry at 3 dpi precipitate a robust neuroinflammatory cascade in the OB, characterized by astrocyte activation and oxidative stress, which likely underlies the early olfactory deficits observed prior to motor symptom onset.

### 3.4 Assessment of adenosine neuromodulatory system in the olfactory bulb

Adenosine modulates synaptic transmission within the OB and regulates barrier integrity in the CNS. To determine whether dysregulated purinergic signaling contributes to early olfactory deficits and EAE pathology, we measured total protein levels and mapped the cellular distribution of the A_1_R, A_2A_R and the principal ectoenzyme generating extracellular adenosine, CD73, in the OB at 3 dpi.

**Figure 8:**
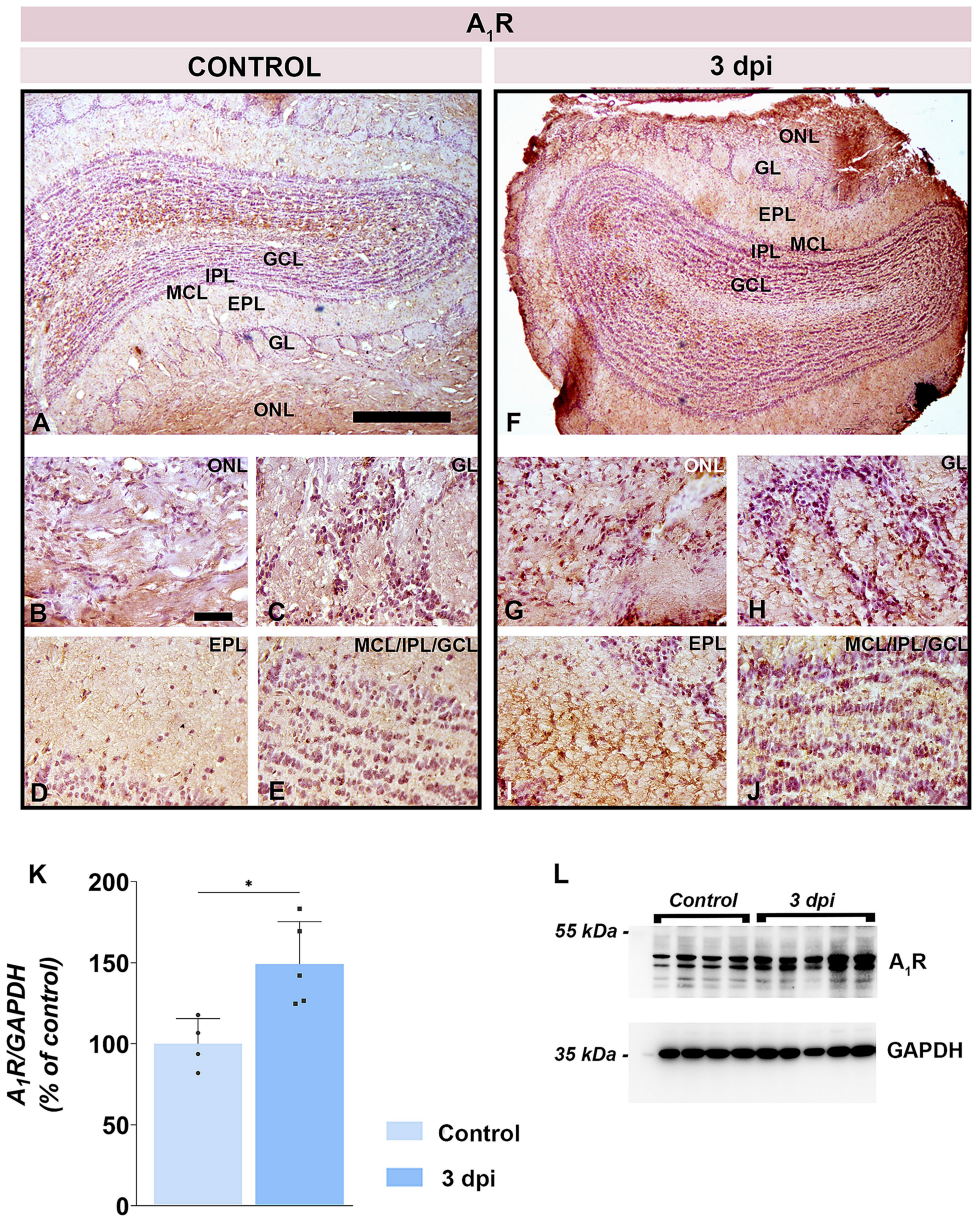
A_1_R expression in the OB. **(A–E)** Representative A_1_R-stained OB sections from control animals. **(F–J)** Representative OB sections from EAE animals at 3 dpi, showing enhanced A_1_R-immunoreactivity. ONL, olfactory nerve layer; GL, glomerular layer; EPL, external plexiform layer; MCL, mitral cell layer; IPL, internal plexiform layer; GCL, granule cell layer. Scale bars: 500 μm **(A,F)**; 50 μm **(B–E,G–J)**. **(K)** A_1_R protein abundance in OB tissue. A_1_R levels were quantified by immunoblotting in OB tissue from control and EAE animals at 3 dpi, using GAPDH as a loading control. A_1_R/GAPDH ratio in EAE animals was normalized to control value arbitrarily set to 100%. Bars represent mean relative abundance ± SD, based on 4 samples per group (P2), analyzed in two independent replicates. Statistical significance: *p < 0.05. **(L)** Representative PVDF membrane showing A1R and GAPDH protein bands.

Western blotting revealed a significant increase in A_1_R abundance at 3 dpi (149.2 ± 26.2% of control; *p* = 0.0203) (Figures 8K,L). Immunohistochemistry confirmed robust upregulation across all OB layers at 3 dpi (Figures 8F–J), with the strongest A_1_R immunoreactivity along the pial surface (Figure 8F) and in the superficial olfactory nerve layer (ONL) (Figure 8G). Enhanced A_1_R staining was also seen in neuronal somata of GL (Figure 8H) and in tuft bodied cells and synaptic puncta within the EPL (Figure 8I), whereas control sections showed only sparse labeling (Figures 8A–E).

**Figure 9:**
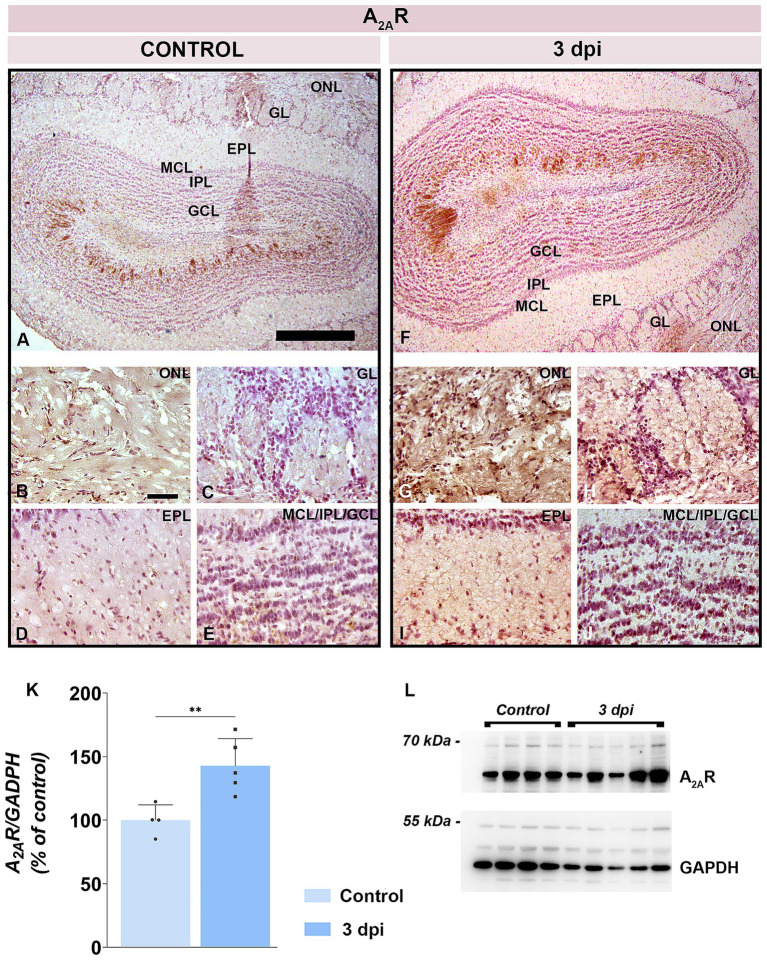
A_2A_R expression in the OB. **(A–E)** Representative A_2A_R-immunostained OB sections from control animals. **(F–J)** Representative OB sections from EAE animals at 3 dpi, showing increased A_2A_R-immunoreactivity. Abbreviations: ONL—olfactory nerve layer; GL, glomerular layer; EPL, external plexiform layer; MCL, mitral cell layer; IPL, internal plexiform layer; GCL, granule cell layer. Scale bars: 500 μm **(A,F)**; 50 μm **(B–E,G–J)**. **(K)** A_2A_R protein abundance in OB tissue. A_2A_R levels were assessed by immunoblotting in OB samples from control and EAE animals at 3 dpi. Protein abundance was normalized to GAPDH as a loading control, and A_2A_R/GAPDH ratios in control samples were arbitrarily set to 100%. Bars represent mean relative abundance ± SD from four samples (P2) per group, analyzed in two independent experiments. Statistical significance: **p < 0.01. **(L)** Representative PVDF membrane showing A_2A_R and GAPDH protein bands.

Total A_2A_R protein level rose to 135.6 ± 20.0% at 3 dpi in respect to control (*p* = 0.0059) (Figures 9K,L). A_2A_R immunolabeling (Figures 9A–J) indicated an increased number of A_2A_R-positive cells at 3 dpi in both the ONL (Figure 9G) and GL (Figure 9H), along with intensified synaptic reactivity in the EPL (Figure 9I). Deeper layers (IPL and GCL) exhibited minimal change (Figure 9J).

**Figure 10:**
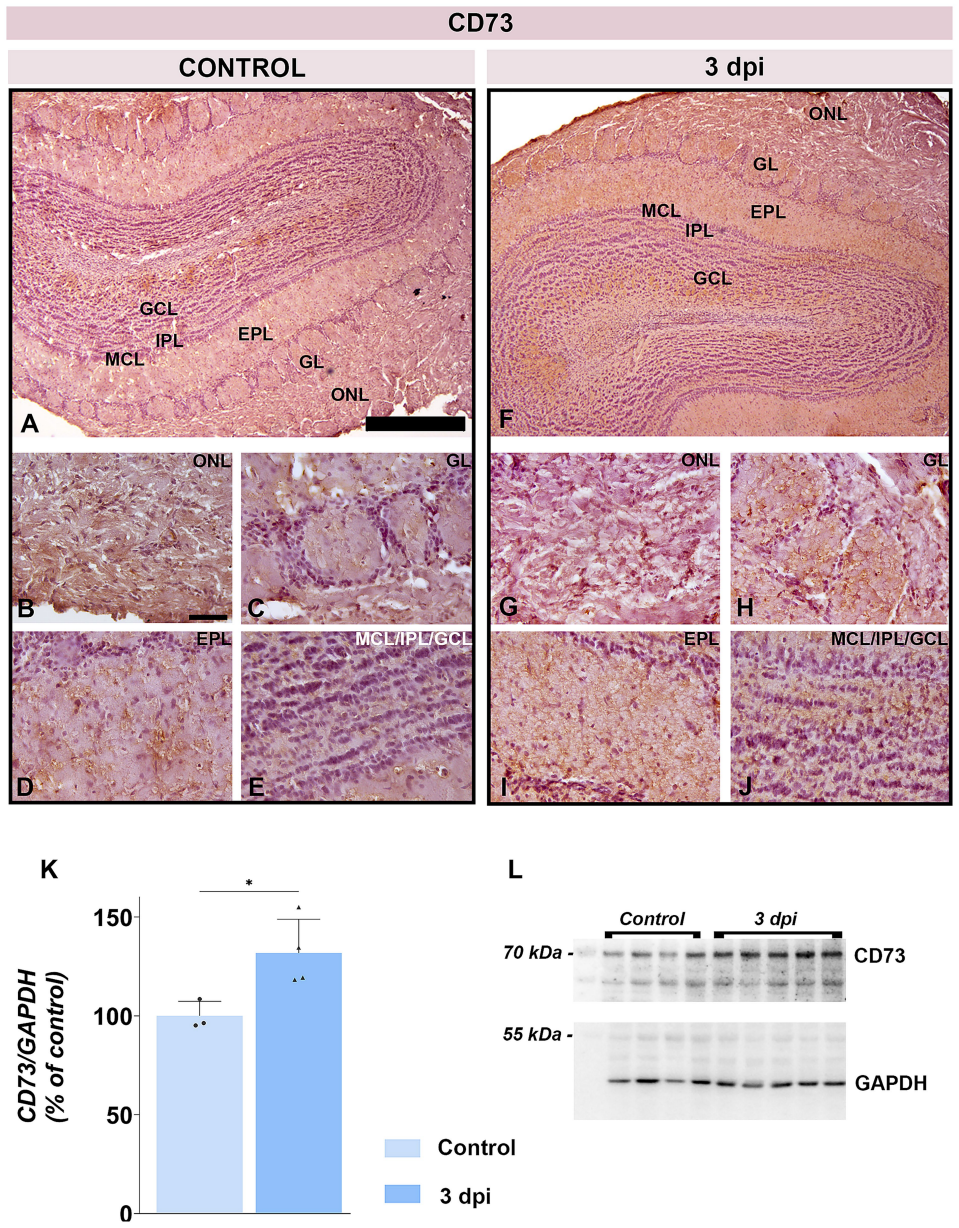
CD73 expression in the OB. **(A–E)** Representative CD73-immunostained OB sections from control animals. **(F–J)** Representative OB sections from EAE animals at 3 dpi, showing increased CD73 immunoreactivity. ONL, olfactory nerve layer; GL, glomerular layer; EPL, external plexiform layer; MCL, mitral cell layer; IPL, internal plexiform layer; GCL, granule cell layer. Scale bars: 500 μm **(A,F)**; 50 μm **(B–E,G–J)**. **(K)** CD73 protein abundance in OB tissue. CD73 levels were measured by immunoblotting in OB tissue from control and EAE animals at 3 dpi. Protein abundance was normalized to GAPDH as a loading control, and CD73/GAPDH ratios in control samples were arbitrarily set to 100%. Bars represent mean relative abundance ± SD from four samples (P2) per group, analyzed in two independent experiments. Statistical significance: *p < 0.05. **(L)** Representative PVDF membrane showing CD73 and GAPDH protein bands.

CD73 protein abundance was also elevated at 3 dpi (131.8 ± 17.15% of control; *p* < 0.05) (Figures 10K,L). CD73 immunoreactivity intensified at 3 dpi throughout the ONL parenchyma (Figure 10G) and in astrocytic processes spanning the GL (Figure 10H), EPL (Figure 10I), and GCL (Figure 10J) in respect to control (Figures 10A–E). The pial surface remained a prominent site of CD73 immunoreactivity in both control (Figure 10A) and EAE (Figure 10F) tissue.

## 4 Discussion

Olfactory dysfunction is among most common manifestations in MS patients (Kim et al., 2018; Lucassen et al., 2016; Mirmosayyeb et al., 2022) progressing to severe olfactory impairment with disease progression (Todd et al., 2023). A recent meta-analysis (Mirmosayyeb et al., 2022) estimated olfactory dysfunction prevalence in MS patients at 11–93%, reflecting methodological differences and underreporting due to patient unawareness (Lucassen et al., 2016; Bsteh et al., 2019; Doty et al., 1997). MRI studies have detected olfactory system abnormalities from early disease stages (Atalar et al., 2018), while lack of adult neurogenesis and impairment along the rostral migratory stream (Tepavčević et al., 2011) shed additional light to higher susceptibility of olfactory system in MS/EAE. Although prior reports document neuroinflammation in the OB during full-blown EAE (Stekic et al., 2024), present study is the first to demonstrate that olfactory impairment emerges as an early non-motor sign in EAE, manifesting several days before classical motor deficits. In present study, more than two-thirds of EAE animals showed significantly weaker performance in BFT as early as ~3 dpi, whereas the first motor deficits appeared at ~7 dpi and severe motor disabilities at ~12 dpi. This temporal gap highlights the OB as one of the first CNS regions targeted by neuroinflammation and suggests olfactory testing as an accessible early biomarker of disease onset.

Importantly, early olfactory impairment in EAE cannot be attributed to motor impairment, since general locomotion and overall mobility in the open-field test remained intact at 3 dpi. Yet, EAE rats exhibited anxiety-like behaviors, spending more time at the periphery in immobile state, with heightened freezing responses and reduced exploration, which mirror early affective changes reported in both MS patients (Margoni et al., 2023; Ellwardt et al., 2022) and EAE (Takahashi et al., 2021; Glinka et al., 2012). In humans, anxiety often precedes motor symptoms by years and correlates with later cognitive decline and memory deficits (Disanto et al., 2018; Ribbons et al., 2017; Kalron et al., 2018), which are clearly related to neurodegeneration (Thomas et al., 2020). The clinical data support a model in which olfactory and affective disturbances reflect early limbic-circuit neuroinflammation intrinsic to MS pathology, rather than secondary response to disability.

It is well established that MS develops as a consequence of multifocal breaches of BBB and CSF–blood barriers, allowing perivascular and subependymal infiltration of peripheral immune cells (Stavropoulos et al., 2021; Balasa et al., 2021; Bennett et al., 2010). Our barrier-integrity assay revealed early, yet transient opening of OB tissue barriers. Specifically, intravenously administered FITC-dextran infiltrated superficial OB layers at 3–5 dpi showing lack of tissue barriers integrity, then normalized by 7 dpi. The breach of the tissue barrier in the OB was confirmed by detecting peripheral monocyte/macrophage infiltrates at 3 dpi, with a lateral-to-medial gradient that paralleled microglial activation. The density of peripheral infiltrates decreased from the superficial layer on the lateral aspect of OB toward deeper OB layers, with no infiltrates detected in the subependymal zone. Accordingly, spatial arrangement of reactive microglia followed the same gradient, with strongly activated microglia in the ONL on the lateral aspect of OB and only slightly activated microglia in the IPL. The superficial ONL hosts dense population of microglia that prevents foreign particles from penetrating the brain. This local population of microglial cells shows more rapid and more intense activation relative to microglia in other regions of the brain, lasting for months after the initial stimuli (Lalancette-Hébert et al., 2009). Reactive microglia release proinflammatory cytokines and produce reactive oxygen (ROS) and nitrogen (NOS) species that damage cells and drive further neuroinflammation. Indeed, we have found substantial neuroinflammatory reaction and oxidative imbalance in OB. In particular, already at 3 dpi OECs and astrocytes across all OB layers showed signs of activation and hypertrophy. These cells are known to play dual roles in neuroinflammation, contributing both to neuroprotection and to the exacerbation of pathology depending on the context and severity of activation, while their involvement in the tissue barrier(s) integrity and inflammatory signaling makes them key players in the early stages of EAE (Liu et al., 2024; Cheng et al., 2014). OB was also affected by significant disturbance in redox homeostasis. Elevated levels of MDA, NO, and O^2−^ at 3 dpi and the concurrent depletion of antioxidant defense systems, including reduced GSH, SH, and tSOD activity, imply oxidative imbalance that contributes to early neuronal dysfunction. While pro-oxidative marker levels returned to control levels by 12 dpi, the oxidative defense systems remained impaired at 12 dpi. These findings indicate that pro-oxidative marker overproduction contributes to early pathogenesis in the OB but does not contribute to advanced disease stages, characterized by myelin loss and axonal damage. On the other hand, persistent suppressions of antioxidant defense systems suggest that oxidative imbalance is a sustained pathological feature in OB, likely contributing to disease progression in MS/EAE (Dasgupta et al., 2013; Qi et al., 2006).

Present study also observed a transient increase in serum neurofilament light chain (NEFL) at 3 dpi, coincident with barrier breach, followed by normalization despite ongoing olfactory and motor deterioration. NEFL is the structural component of axon-specific intermediate cytoskeletal elements, released into the bloodstream as a result of axonal damage (Gaetani et al., 2019; Kuhle et al., 2017). Persistently elevated serum NEFL levels are found in MS patients due to both acute localized inflammatory activity and chronic brain-diffuse neurodegeneration (Gaetani et al., 2019; Yuan et al., 2017; Kuhle et al., 2017). Transient surge of NEFL serum levels is in accordance with acute barrier disruption in EAE, which allows limited detection of neuroaxonal injury peripherally, although chronic oxidative and inflammatory processes drive the ongoing neurodegeneration.

Our mapping of adenosine signaling components reveals a pivotal role in early EAE pathogenesis, with implications for both sensory dysfunction and CNS barrier modulation. Adenosine critically regulates both synaptic transmission within the OB (Rotermund et al., 2019; Rotermund et al., 2018), inflammation (Kozic et al., 2025), and the integrity of the CNS tissue barriers (Bynoe et al., 2015). Present study demonstrates concurrent upregulation of CD73, the key ectoenzyme generating extracellular adenosine, and principal adenosine receptors, A_1_R and A_2A_R, across OB layers at 3 dpi.

Several recent studies demonstrate that enhanced adenosine signaling correlates with enhanced BBB and CSF–blood barrier gateway and T-cell infiltration across the tissue barriers in EAE (Zheng et al., 2022; Bynoe et al., 2015). At 3 dpi, CD73 and A_1_R/A_2A_R were upregulated across OB layers, especially along the pial surface and ONL, implying that locally amplified adenosine signaling may be critically involved in early barrier regulation at the level of OB. Simultaneous upregulation of A_1_R and A_2A_R aligns with prior evidence that both receptor subtypes are involved in tissue barrier permeability. Overexpression and activation of A_2A_Rs are critical for endothelial and epithelial cell tight junction disruption in neuroinflammatory conditions (Zheng et al., 2022; Nedeljkovic, 2019), facilitating immune cell infiltration in brain parenchyma. With regard to A_1_R, although enhanced signaling is viewed as neuroprotective, recent studies suggest that it has a permissive role in CNS barrier opening when co-activated with A_2A_R. Specifically, evidence suggests that increased A_1_R activation increases the cell surface expression of A_2A_R (Stockwell et al., 2017). Robust co-expression of A_1_R, A_2A_R, and CD73 along the pial surface and in the ONL, particularly as these sites are positioned at the interface between the periphery and the CNS, may serve as entry points for peripheral immune cells in neuroinflammatory conditions.

Finally, when evaluating the results of the present study, it is particularly important to point out the spatial pattern of the observed responses, from FITC-dextran fluorescent penetration, distribution of peripheral infiltrates, and the pattern of microglial activation in the OB. In particular, FITC fluorescence was confined strictly on the lateral aspect of OB, which is not consistent with dextran diffusion throughout the leaking vasculature wall. The fluorescent signal was also absent from the centrally located subependymal zone which is directly adjacent to the narrow olfactory ventricle connected to cerebral ventricle. These observations argue against substantial immune cell entry from bloodstream or CSF-filled ventricular system at least at the early stage of EAE and are more consistent with their entry from the subarachnoid space, through arachnoid and pial membranes. Recent study reporting comprehensive spatiotemporal assessment of CNS infiltrations in EAE indeed demonstrated that the SAS serves both as the site for precursor CD4^+^ T-cell proliferation and as their gateway to CNS parenchyma (Brown and Sawchenko, 2007). Upon induction, autoreactive CD4^+^ T cells accumulate within regional lymph nodes from where they easily access the leptomeninges and enter the SAS (Shin et al., 1995). Moreover, cranial and spinal nerve root sheaths are permeable to macromolecules, which explain why inflammatory cell aggregates are often found around nerve roots in the very early stage of EAE (Shin et al., 1995). It appears, however, that olfactory nerve and cervical lymph nodes play critical role in EAE induction (Laman and Weller, 2013), due to unique meningeal architecture and lymphatic drainage at the level of OB. Anatomical discontinuities in the arachnoid membrane on the CNS side of the cribriform plate (Fatuzzo et al., 2023; Spera et al., 2023) allow a substantial fraction of CSF to bypass arachnoid granulations, flowing instead along perineural routes through the cribriform plate and along olfactory nerve into the nasal submucosa (Laaker et al., 2023; Zakharov et al., 2004). From there, CSF-borne antigens enter nasal lymphatics and drain to deep cervical lymph nodes, a conduit critical for priming autoreactive CD4^+^ T cells (Zakharov et al., 2004). This nasal lymphatic outflow and olfactory nerve establish a direct link between CSF dynamics, lymph node activation, and early OB inflammation, shedding light on why olfactory dysfunction emerges so early in MS/EAE and other neurodegenerative diseases.

Collectively, our findings position the OB as a neuroimmune interface and permissive entry zone in the earliest stages of EAE. The unique meningeal and lymphatic anatomy at the cribriform plate may explain why olfactory dysfunction is a common, early feature of MS/EAE and highlight olfactory and behavioral assessments as valuable tools for early MS detection and monitoring. These findings suggest that olfactory impairment may serve as an early indicator of CNS pathology in autoimmune demyelination and could allow earlier diagnosis and faster therapeutic intervention, which is critical for improving treatment outcomes. Although EAE recapitulates important features of MS, it does not fully reflect the complexity of the human disease, especially the heterogeneity of clinical presentation and disease courses. Therefore, future studies combining behavioral, imaging, and biomarker analyses in both animal models and MS patients will be essential to validate olfactory dysfunction as a clinically useful early biomarker for MS.

## Data Availability

The original contributions presented in the study are included in the article/Supplementary material, further inquiries can be directed to the corresponding author.
