# Supplementary material for: Early olfactory dysfunction in experimental autoimmune encephalomyelitis reflects transient brain barrier breach and initiation of neuroinflammation in the olfactory bulb
Source: Front Cell Neurosci. 2025 Sep 3;19:1656777. doi: 10.3389/fncel.2025.1656777 (PMC12442833; doi:10.3389/fncel.2025.1656777)
Supplement: Supplementary file 1 [file Table_1.DOCX]

Supplementary Material

Early olfactory dysfunction in experimental autoimmune encephalomyelitis reflects transient brain barrier breach and initiation of neuroinflammation in the olfactory bulb

**Andjela Stekic^1^, Milorad Dragic^1,3^, Ivana Stevanovic^2^, Marina Zaric Kontic^3^, Marija Adzic Bukvic^1^, Sanja Dacic^1^, Milica Ninkovic^2^, Nadezda Nedeljkovic^1^***

^1^Center for Translational Neuroscience, Department of General Physiology and Biophysics, Faculty of Biology, University of Belgrade, Belgrade, Serbia

^2^Medical Faculty of Military Medical Academy, University of Defense, Belgrade, Serbia

^3^Department of Molecular Biology and Endocrinology, Vinca Institute of Nuclear Sciences-National Institute of the Republic of Serbia, University of Belgrade, Belgrade, Serbia

*** Correspondence:** Nadezda Nedeljkovic: [nnedel@bio.bg.ac.rs](mailto:nnedel@bio.bg.ac.rs)

# Supplementary Figures and Tables

## Supplementary Figure *S1*


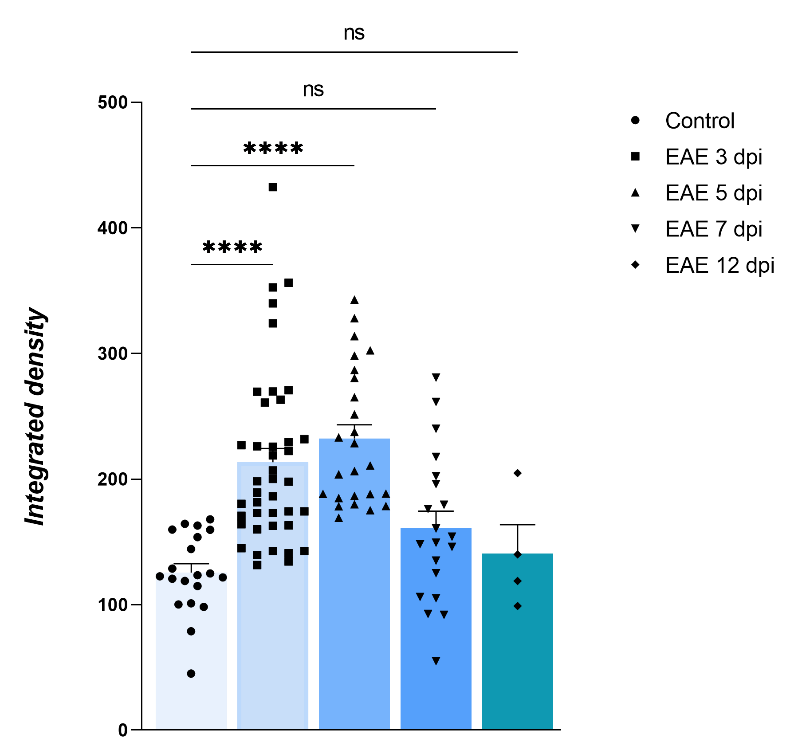


**Supplementary Figure *S1*.** **Quantification of FITC-dextran leakage analysis.** Quantitative analysis was expressed as integrated density of the fluorescent signal in 5 × magnification micrographs at 3, 5, 7 and 12 dpi in comparison with control. Bars represent mean integrated density ± SEM from n > 5 microscopic frames from 4 different sections.

**2 Supplementary Tables**

**2.1 Supplementary Table S1**

| **Supplementary Table *S1***. **Epidemiological data** | | |  |
| --- | --- | --- | --- |
| **Parameter** | | **Control** | **EAE** |
| Total number of animals | | 90 | |
| No. of animals in experimental group | | 22 | 68 |
| Number of experimental units | | 5 | 17 |
| Animals excluded from the study | | 0 | 2 |
| Mean body mass at the begining of study (g) | | 200.6 ± 6.3 | 187.8 ± 19.8 |
| Mean body mass at sacrifice (g) | | 200.1 ± 14.6 | 181.1 ± 19.2 (3 dpi)  180.8 ± 19.8 (5 dpi)  181.9 ± 24.9 (7 dpi)  152.3 ± 18.3 (~12 dpi) |
| Incidence of EAE | | / | 66/68 (97 %) |
| Mean dpi to onset | | / | ~7 dpi |
| Mean dpi to sacrifice | |  | ~12 dpi |
| Mean dpi to sacrifice in early phase of EAE | | / | 3* |
| Incidence of OD at 3 dpi | | / | 69.1 % |
| Maximum mean score (at ~12 dpi) | | / | 2.1 ± 1.2 |
| Mortality | | / | 0/68 |
| Latency in BFT (s) | | 88.0 ± 29.9 (baseline) | 269.0±62.8 (3 dpi) |

**All animals that exhibited OD at 3 dpi were sacrificed that day.*
